# Sustainable strategies for management of the “false root-knot nematode” *Nacobbus* spp.

**DOI:** 10.3389/fpls.2022.1046315

**Published:** 2022-11-25

**Authors:** Paola Lax, María A. Passone, Alejandra G. Becerra, Ana L. Sosa, Aurelio Ciancio, Mariella M Finetti-Sialer, Laura C. Rosso

**Affiliations:** ^1^Instituto de Diversidad y Ecología Animal (Consejo Nacional de Investigaciones Científicas y Técnicas-Universidad Nacional de Córdoba), Facultad de Ciencias Exactas, Físicas y Naturales, Universidad Nacional de Córdoba (UNC), Córdoba, Argentina; ^2^Centro de Zoología Aplicada, Facultad de Ciencias Exactas, Físicas y Naturales, Universidad Nacional de Córdoba (UNC), Córdoba, Argentina; ^3^Laboratorio de Ecología Microbiana Ambiental (ECOMA), Facultad de Ciencias Exactas, Físico-Químicas y Naturales, Universidad Nacional de Río Cuarto (UNRC), Rio Cuarto, Argentina; ^4^Instituto Multidisciplinario de Biología Vegetal (Consejo Nacional de Investigaciones Científicas y Técnicas-Universidad Nacional de Córdoba), Facultad de Ciencias Exactas, Físicas y Naturales, Universidad Nacional de Córdoba (UNC), Córdoba, Argentina; ^5^Consiglio Nazionale delle Ricerche, Istituto per la Protezione Sostenibile delle Piante, Bari, Italy; ^6^Consiglio Nazionale delle Ricerche, Istituto di Bioscienze e Biorisorse, Bari, Italy

**Keywords:** *Nacobbus* spp., nematode-plant interaction, biological control, eco-compatible strategies, resistance

## Abstract

The genus *Nacobbus*, known as the false root-knot nematode, is native to the American continent and comprises polyphagous species adapted to a wide range of climatic conditions. Alone or in combination with other biotic and abiotic factors, *Nacobbus* spp. can cause significant economic yield losses on main food crops such as potato, sugar beet, tomato, pepper and bean, in South and North America. Although the genus distribution is restricted to the American continent, it has quarantine importance and is subject to international legislation to prevent its spread to other regions, such as the European Union. The management of *Nacobbus* spp. remains unsatisfactory due to the lack of information related to different aspects of its life cycle, survival stages in the soil and in plant material, a rapid and reliable diagnostic method for its detection and the insufficient source of resistant plant genotypes. Due to the high toxicity of chemical nematicides, the search for alternatives has been intensified. Therefore, this review reports findings on the application of environmentally benign treatments to manage *Nacobbus* spp. Biological control strategies, such as the use of different organisms (mainly bacteria, fungi and entomopathogenic nematodes) and other eco-compatible approaches (such as metabolites, essential oils, plant extracts, phytohormones and amendments), either alone or as part of a combined control strategy, are discussed. Knowledge of potential sources of resistance for genetic improvement for crops susceptible to *Nacobbus* spp. are also reported. The sustainable strategies outlined here offer immediate benefits, not only to counter the pathogen, but also as good alternatives to improve crop health and growth.

## The genus *Nacobbus*

The genus *Nacobbus* (family Pratylenchidae) comprises plant-parasitic nematode (PPN) species causing the formation of galls on roots in susceptible hosts. This endoparasite is known as the “false root-knot nematode” because the symptoms of the attacked roots are similar to those induced by root-knot nematodes, *Meloidogyne* spp. The genus occurs in temperate and subtropical latitudes of South and North America, with *N. aberrans* as first described species. Sher (1970) reviewed the genus and analyzed the type material, recognizing *N. dorsalis* and *N. aberrans* as the only valid species. Despite the clear differentiation between the two species, as new populations of *N. aberrans sensu* Sher were detected in the American continent, a marked variability became evident, mainly at morphological, physiological and genetic levels, generating controversy about the genus taxonomy (Lax et al., 2021). For this reason, the term *N. aberrans sensu lato* has been commonly used for the complex which would comprise species that are very difficult to distinguish morphologically.

Molecular analyses (ITS rDNA) supported the hypothesis that *N. aberrans s.l.* would comprise a species complex (Reid et al., 2003; Lax et al., 2014). A recent integrative taxonomy analysis, carried out by using morphometric and molecular data (Lax et al., 2021), supported the identification of three nominal species: *i) N. aberrans sensu stricto*, mainly distributed in Mexico and Ecuador; *ii) N. bolivianus*, present in Bolivia and Peru; *iii) N. celatus*, a new taxon widely distributed in the lowlands of Argentina. The complex still remains to be exhaustively resolved because there are many populations, especially in South America, that could represent new taxa (Lax et al., 2021). In the present review, the term *Nacobbus* species complex (NSC) includes *N. aberrans s.s.*, *N. celatus*, *N. bolivianus* and *Nacobbus* sp. The populations previously reported as *N. aberrans* and whose identity has not been corroborated on the basis of morphological and molecular studies are referred to as *N. aberrans s.l.*

### Life cycle and parasitism

**Figure 1:**
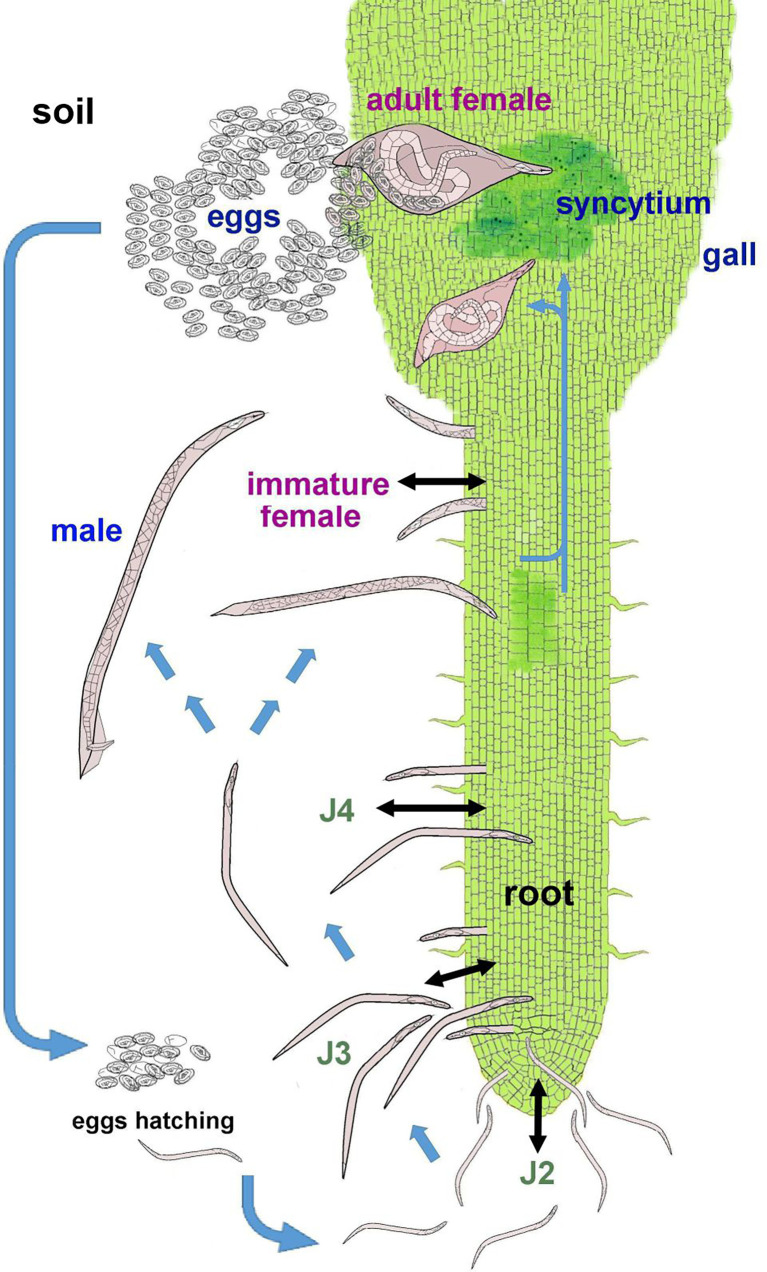
Life cycle of *Nacobbus* spp. Blue arrows indicate the development of the different stages through the life cycle; black arrows represent the movement of the infective stages, both in soil and in roots. In soil, the J2 hatches from the egg and then molts to J3 and J4. These stages enter the root, migrate causing necrosis and can also leave the host tissues. The J4 molts again to a male or immature female. The female invades the root, migrates in proximity of the central cylinder and causes histological alterations that induce the feeding site (syncytium) and the formation of the gall. The female body swells as she feeds. Once fertilized by the male, the female produces an egg mass, which is laid on the gall in contact with soil particles.

The *Nacobbus* reproductive biology is likely sexual although it may be facultative parthenogenetic (Reid et al., 2003). The life cycle includes the embryonated egg, four juvenile stages (J1 inside the egg, J2-J4) and adults (male and female) **(** **Figure 1****)**. *Nacobbus* spp. are the only known PPN with an unusual cycle including infective and migratory endoparasitic stages (J2-J4 and immature female), with a further sedentary stage (mature female). The J2-J4 and the migratory female can be found in soil and also in the root, where they move intracellularly and destroy tissues, causing necrotic lesions. These stages leave and re-invade host roots, producing additional damage. The males, found in soil or roots, leave the host after molting, searching for females. Mature females are sedentary and feed, inducing physiological alterations (hyperplasia and hypertrophy) in roots, resulting in the formation of the feeding site (syncytium), inside the gall. The fertilized female deposits eggs on the gall surface in a mucilaginous egg mass. The life cycle can be completed in about 37-48 days at 22-24°C (Costilla, 1985a). The cycle is influenced by several factors, with host and temperature as the most important, the latter affecting developmental processes such as embryogenesis, egg hatching (Clark, 1967; Inserra et al., 1983), J2 root penetration and sex ratio (Prasad and Webster, 1967).

Due to their great adaptive capacity, *Nacobbus* spp. are found in very different environments, ranging from sea level (Doucet and Lax, 2005) to the Andean highlands (about 4,000 m a.s.l.) (González and Franco, 1997). The NSC shows a preference for drier climates. Conversely, warm and wet areas do not appear to be very favorable for parasite development (Costilla, 1985a). The quiescence and diapause of eggs and other stages play a very important role for survival under adverse conditions (Manzanilla-López et al., 2002), such as low temperatures (-20° C) and desiccation for prolonged periods of time (Costilla, 1985a; González and Franco, 1997). The J4 is the stage best adapted to survival, given its higher lipid reserve (Souza and Baldwin, 2000). The adaptive capacity of this nematode is also reflected in its efficient dispersal mechanism, mainly through passive transport by contaminated tubers.

**Figure 2:**
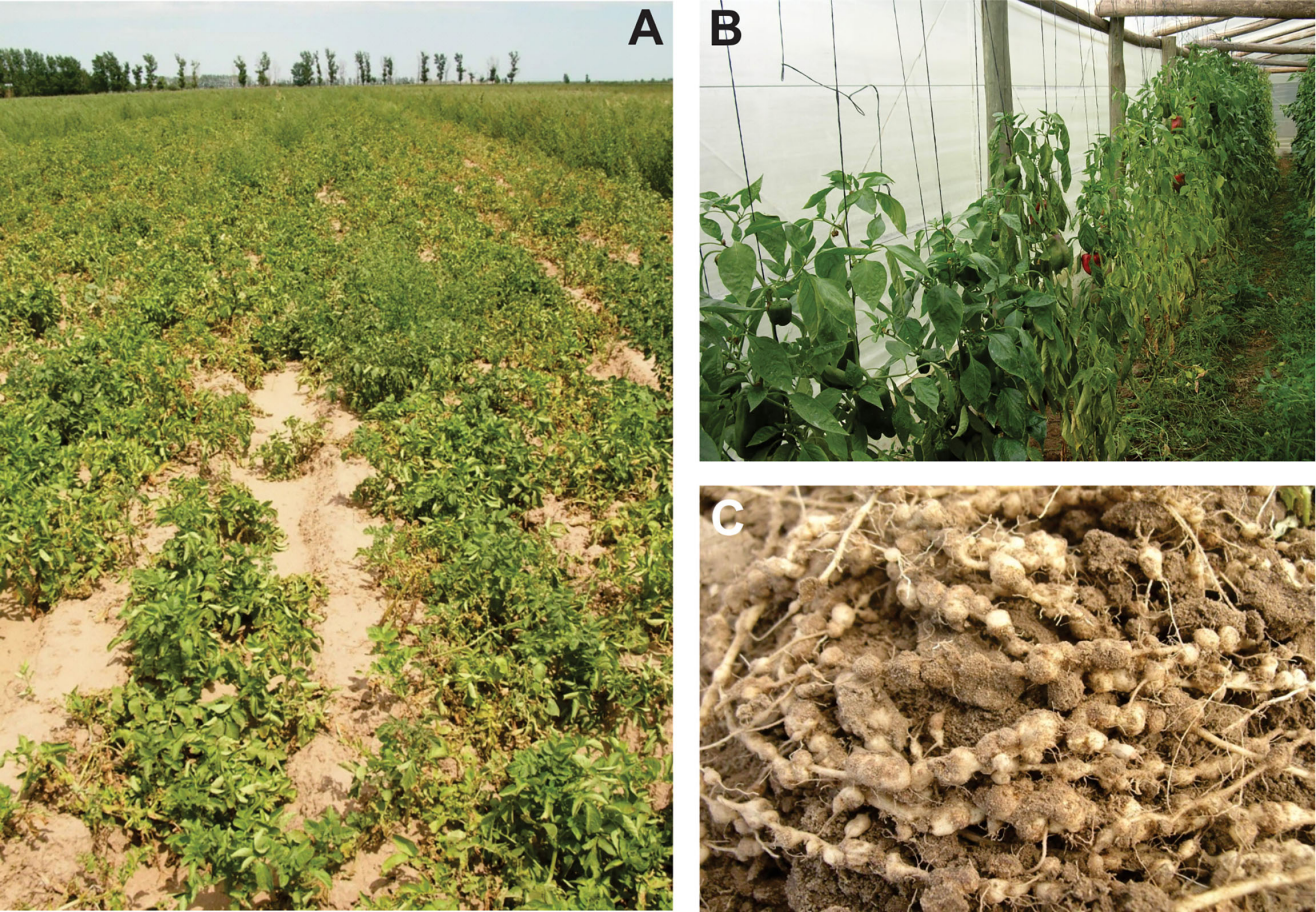
Symptoms caused by *Nacobbus* spp. **(A)** Potato crop with patches due to the presence of *Nacobbus* sp.; **(B)** Pepper crop under greenhouse conditions, showing plants with poor development and wilting due to *N. celatus* attack; **(C)** Chard roots with numerous galls induced by *N. celatus*.

The symptoms caused by *Nacobbus* spp. can be shown not only in the root system (galls) but also in the aerial part of the host plant (**Figure 2**). In highly infested fields it is possible to observe areas or patches with poor plant growth, stunting, chlorosis and signs of wilting (Cabrera Hidalgo et al., 2014). Even in severe attacks, the host may die. A parasitised plant has a lower chance of resisting unfavorable conditions, especially drought (Costilla, 1985a). Damages can be even greater when the nematode coexists with other pathogens, such as fungi (Rodríguez et al., 2007) or other PPN species (Lax et al., 2006a; Ortuño et al., 2013).

**Figure 3:**
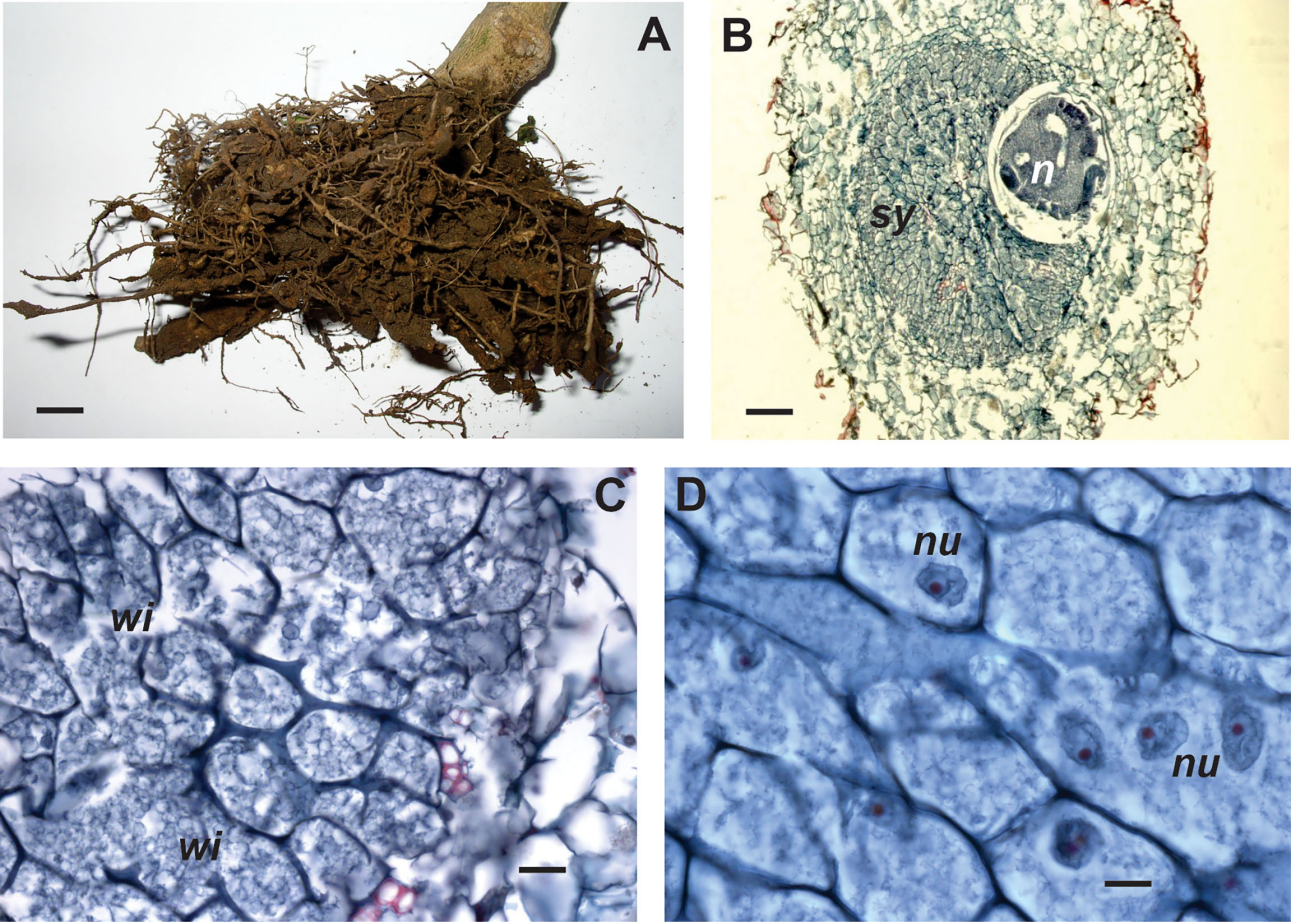
External view and anatomy of galls induced by *Nacobbus celatus* in different pepper cultivars. **(A)** Cv. Yatasto with poor root development due to severe nematode attack and some galls. Histological cross-sections of gall. **(B)** Cv. California wonder, syncytium (*sy*) located in the central cylinder and body female (*n*); **(C)** Cv. Yatasto, detail of syncytial cells with dense cytoplasm, thickened walls and few wall interruptions (wi); **(D)** Cv. Fyuco, detail of syncytial cells with several nuclei (*nu*) of different shape and size. Scale bars: A = 5 mm; B = 100 μm; C = 20 μm; D = 10 μm.

The infected roots show galls of varying size (approximately 2-5 mm), with abundant lateral root proliferation. Inside the gall (**Figure 3**), the anterior region of the swollen female is embedded in the syncytium that is composed of cells (more than 30 in a transverse section) with variable shape and different degrees of hypertrophy (Lax et al., 2013a). The feeding site develops either in the cortex or in the central cylinder (Moyetta et al., 2007), producing a disorganization, displacement, and fragmentation of vascular tissues (Tordable et al., 2010a). Syncytium is composed of hyperplastic cells, parenchymatic cells of vascular tissues, phloem and xylem, and cells of the vascular cambium (Tordable et al., 2010b). Syncytial cells have dense, vacuolated and fibrillar cytoplasm, hypertrophied nuclei, evident nucleoli and thickened cellulosic walls, partially fragmented (Tordable et al., 2018). Starch granules are characteristic of NSC syncytia (Souza, 2001; Cabrera et al., 2017).

### Economic importance

NSC is widespread in the American continent with a significant economic impact, being one of the top 10 most important PPN (Jones et al., 2013). It has quarantine importance and is subject to international legislation to prevent its spread to other regions, such as the European Union (EFSA Panel on Plant Health et al., 2018). Although quarantine records and interceptions of contaminated plant material have been reported in other countries (e.g., England, the Netherlands and Russia), there is no evidence so far of its establishment outside the American continent (EPPO, 2022).

*Nacobbus* spp. are highly polyphagous, attacking at least 84 plant species of 24 families, including weeds, native plants and crops (Manzanilla-López et al., 2002; Doucet and Lax, 2005; EPPO, 2009). The main hosts are tomato (*Solanum lycopersicum*), potato (*S. tuberosum*), sugarbeet (*Beta vulgaris*), pepper (*Capsicum annuum*), and bean (*Phaseolus vulgaris*). *Nacobbus* populations from distinct geographical origins may exhibit different behavior on the same plant species or cultivar, showing that some of them have the ability to invade the roots and multiply while others are not able to parasitise them. Based on that host preferences, populations can be differentiated into groups or pathotypes (Castiblanco et al., 1999; Manzanilla-López et al., 2002; Inserra et al., 2005; Lax et al., 2011a) that are associated with a certain host range and geographic area (EFSA Panel on Plant Health et al., 2018) (**Table 1**). That variability of field populations complicates nematode control. However, so far, there is no consensus or a definitive test for this physiological classification as occurs in other PPN species (Lax et al., 2011a).

**Table 1:** Host range and distribution of physiological groups of *Nacobbus* spp. based on host preference.

Group*	Range host	Distribution	Species
Bean	Bean and pepper but not potato or sugarbeet	Mexico	*N. aberrans s.s.*
Potato	Potato, sugarbeet and tomato but not pepper	Andean regions of Argentina, Bolivia, Chile and Peru Lowlands of Argentina and Mexico	*Nacobbus* sp. *N. aberrans s.s.* *N. bolivianus* *N. celatus*
Sugarbeet	Sugarbeet, pepper and tomato but not potato	Lowlands of Argentina, USA, and Ecuador	*N. aberrans s.s.* *N. celatus*

*Classification based on Manzanilla-López et al. (2002).

There is little information on estimates of NSC damage and economic threshold. The nematode represents a potential risk for different horticultural crops due to the wide host range. The impact on yield depends on different factors, such as nematode population, initial density, climatic conditions, soil type, and the crop/cultivar selected. The reproduction is higher at low initial populations due to reduced competition for penetration sites and greater food availability (Cusicanqui et al., 1997). Even in incipient populations (1-15 individuals/100 g soil), the nematode can significantly affect the crop yield (33% losses) (Franco et al., 1999). In the USA, losses range 10-20% in sugarbeet (Inserra et al., 2005), and may reach 36% on bean in Mexico (Manzanilla-López et al., 2002). On tomato, NSC limited yields by approx. 60-75% in Ecuador (Corrales Arango, 2007) and 12-83% in Mexico (Cristóbal-Alejo et al., 2006). In the Andean region, *N. aberrans s.l.* is the main pathogen affecting potato in Chile (Franco and Main, 2008), Argentina, Bolivia and Perú (Inserra et al., 1985; Franco, 1994). In the latter two countries, yield is reduced by about 10-73% (Canto-Saenz et al., 1996; Franco et al., 1996), with economic losses that reached 52 millions dollars in Bolivia (Franco et al., 1999). The potato group/pathotype has a particular relationship with this crop because it not only attacks the roots, but also invades the potato skin (under the lenticels) and, in some cases, the tuber parenchyma (Tordable et al., 2018). Quiescent J3 and J4 are able to survive for more than 10 months in stored potatoes; it is also possible to find J2, males, immature females and, rarely, mature females with their egg mass inside the parenchyma. Once a tuber infested with J4 and/or immature females is sown, the cycle is completed in approximately 22 days (Costilla, 1985a). This special nematode-potato interaction generates not only quantitative but also qualitative damage because it affects the seed certification (Franco, 1994), and it requires reliable diagnostic methods to certify nematode-free potato seed (Atkins et al., 2005).

## Sustainable strategies for management

However, the management of the NSC remains limited since reliable methods for its detection are still needed and important aspects of its life cycle, such as the survival capacity of the different stages in soil and/or plant material, are still poorly known. In addition, the formulation of strategies is complicated by field populations belonging to different groups/pathotypes (Franco and Main, 2006), their ability to establish in different environmental conditions and their polyphagy.

The use of conventional nematicides is being progressively restricted due to the damage they cause to health and the environment. For this reason, we focus here on eco-compatible alternatives. Biological control consists of the application of microorganisms to regulate pest population density and/or reduce damage. Biological control agents (BCAs) may interact with the pathogen directly (e.g. through antibiosis, competition for nutrients or space) or indirectly through the host (e.g. induction of resistance) (Poveda et al., 2020). Different beneficial microorganisms (such as bacteria, nematode antagonists, fungi, viruses, and other invertebrates) and/or their metabolites can be used as bio-pesticides. One of the advantages of BCAs is that, in integrated pest management programs, they have shown to be viable when applied in combination and/or with reduced doses of chemicals (Ntalli et al., 2020). The main results of the different BCA-*Nacobbus* spp. interactions are presented in **Table 2** and **Supplementary Table 1**. Other strategies based on eco-compatible approaches are presented in **Table 3** and **Supplementary Tables 2 and 3**. Those sustainable alternatives for control of *Nacobbus* spp. show that their application, alone or as part of a combined control strategy, can improve soil and crop health.

**Table 2:** Response of *Nacobbus* spp. to the interaction with different biological control agents in *in vivo* experiments.

Biocontrol agent (BCA)	*Nacobbus* spp. and origin	Conditions tested	Response to BCA-nematode interaction	Reference
**Bacteria**				
*Pseudomonas protegens* CHA0	N. celatus*; Argentina: Buenos Aires	Tomato cv. Platense, cell suspensions	↓ Galls, egg masses, nematode reproduction No effect on shoot/root weight/length	Lax et al. (2013b)
**Entomopathogenic nematodes/symbiotic bacteria**				
*Steinernema rarum* ACAB, *Heterorhabditis* *bacteriophora* RACA	*N. celatus*; Argentina: Córdoba	Tomato cv. Platense, IJ application	No effect on galls and egg masses ↓ Nematode reproduction	Caccia et al. (2013)
*S. rarum*/*Xenorhabdus szentirmaii*	*N. celatus*; Argentina	Tomato cv. Platense, IJ and SB application	↓ Galls, nematode reproduction	Lax et al. (2013c)
*H. bacteriophora* CBA/*Photorhabdus luminescens* *Steinernema* sp./*Xenorhabdus* sp. *S. rarum* RACA/*X. szentirmaii*	*N. celatus*; Argentina: Córdoba and Tucumán	Tomato cv. Platense, IJ application	No effect on galls, egg masses or nematode reproduction ↑ Biomass only with *H. bacteriophora* CBA-Córdoba	Caccia et al. (2018)
		Tomato cv. Platense, SB suspensions and cell-free supernatants	↑ Biomass with BS suspension of *Xenorhabdus* sp.-Tucumán ↓ Biomass with supernatant of *P. luminescens*-Tucumán Supernatants of *Xenorhabdus* sp. and *P. luminescens*: most effective in ↓ galls, egg masses and nematode reproduction	
**Mycorrhizal fungi**				
*Glomus* sp.	*N. aberrans s.l.*; Mexico	Tomato	↓ Galls	Gardezi et al. (1995)
*Rhizoglomus intraradices*	*N. celatus*; Argentina: Buenos Aires	Tomato cv. Platense	↓ Root gall index ↓ Nematode reproduction No consistent effect on biomass ↑ Mycorrhizal colonization	Lax et al. (2011b)
*R. intraradices*	*N. celatus*; Argentina: Córdoba	Tomato cv. Platense	↓ Galls, nematode reproduction ↑ Shoot and root dry weight ↑ Mycorrhizal colonization	Marro et al. (2014)
*R. intraradices*, *Funneliformis mosseae*	*N. celatus*; Argentina: Córdoba and Tucumán	Tomato cv. Platense, pepper cv. California wonder	↓ Galls, nematode reproduction ↑ Shoot and root dry weight	Marro (2018)
*R. intraradices*, *F. mosseae*	*N. celatus*; Argentina: Tucumán	Tomato cv. Platense	↓ Nematode root invasion	Marro et al. (2018)
*R. intraradices* (B1, A2), *F. mosseae*	*N. aberrans s.l.*; Argentina	Pepper cv. Paco f1	↑ Mycorrhizal colonization ↓ Nematode reproduction ↓ Contents of chlorophyll, carotenoids and soluble proteins	Bernardo et al. (2020)
**Nematophagous fungi**				
*Purpureocillium lilacinum*	*N. aberrans s.l.*; Argentina	Tomato cv. Platense	↓ Galls, nematode reproduction ↑ Egg masses parasitism	Mareggiani et al. (1985)
*P. lilacinum*	*N. aberrans s.l.*	Tomato cv. Jefferson	LD_50_: J2 mortality in soil	Eguiguren-Carrión (1995)
*P. lilacinum* (LPSC# 876)	*N. aberrans s.l.*; Argentina: Buenos Aires	Tomato cv. Platense	No consistent results	Gortari and Hours (2016)
*P. lilacinum* (SR7, SR14, SR38)	*N. celatus*; Argentina: Córdoba	Tomato cv. Platense	↓ Egg masses ↑ Plant growth	Sosa et al. (2021a)
*Pochonia chlamydosporia* (MHCH, SM4, SMB3, SMB3A, SC1, VC10)	*N. aberrans s.l.*; Mexico: Mexico, Puebla and Zacatecas	Tomato cv. Río Grande; bean cv. Canario Criollo	↑ Egg masses and eggs/g root parasitism ↓ Egg hatching	Flores-Camacho et al. (2007)
*P. chlamydosporia* (SC1, IZ1)	*N. aberrans s.l.*; Mexico: Zacatecas	Bean cv. Flor de Mayo Criollo	↓ Galls, nematodes inside roots ↑ Foliage dry and fresh root weight	Franco-Navarro et al. (2012)
*P. chlamydosporia* (MPc1-MPc5)	*N. aberrans s.l.*; Mexico: Puebla	Tomato cv. Río Grande	↓ Galls, nematodes inside roots ↑ Leaf dry weight	Pérez-Rodríguez et al. (2007)
*Fusarium solani* (Fs03), *F. oxysporum* (Fo07, Fo10), *Penicillium janthinellum* (Pe11)	*N. aberrans s.l.;* Mexico	Pepper cv. Yolo Wonder	↓ Galls, J2/g root No consistent effect on growth plant	Cortez-Hernández et al. (2019)

*All *N. celatus* populations were previously identified as *N. aberrans*. J2, second-stage juveniles; SB, symbiotic bacteria; IJ, infective juveniles; LD, lethal dose.

Up and down arrow mean increase and decrease, respectively.

**Table 3:** Response of *Nacobbus* spp. to the interaction with different biorational products (metabolites, essential oils, extracts, phytohormones) in *in vivo* experiments.

Biorational chemical agents	*Nacobbus* spp. and origin	Cultures tested	Action on *Nacobbus*	Reference
**Bacterial metabolite**				
Prodigiosin	N. celatus*; Argentina: Córdoba	Tomato cv. Platense	↓ J2 root invasion	Gomez Valdez et al. (2022)
**Essential oils**				
*Mentha piperita, Laurus nobilis, Eucalyptus globulus, Cinnamomum verum*	*N. aberrans s.l.*; Argentina: Buenos Aires	Tomato cv. Trueno	↓ Number of egg/g root, galls (*M. piperita, E. globulus*) ↑ Total yield (*E. globulus*)	De Lillo (2019)
*M. piperita, L. nobilis, E. globulus*	*N. aberrans s.l.*; Argentina: Buenos Aires	Chard cv. Fordhook	↓ Number of egg/g root	Rípodas (2017)
*Tagetes lucida*	*N. aberrans s.l*.; Mexico	Tomato cv. Río Grande	↓ Galls	Zarate-Escobedo et al. (2018)
**Aqueous extracts**				
*Melia azedarach, E. globulus, Trichilia glauca, Ricinus communis*	*N. aberrans s.l.*; Argentina	Pepper cv. California Wonder	↓ Galls (*M. azedarach*)	Mareggiani et al. (2005)
**Phytohormones**				
Salicylic acid, jasmonic acid, ethylene	*N. aberrans s.l.*; Argentina: Buenos Aires	Tomate cv. Elpida	↓ Galls, nematode reproduction ↑ Total yield	Martinez et al. (2021)

*All *N. celatus* populations were previously identified as *N. aberrans*. J2, second-stage juveniles.

Up and down arrow mean increase and decrease, respectively.

### Bacteria

Plant growth-promoting rhizobacteria colonize roots, and provide several benefits, such as a stimulation of plant growth and pathogens control (Jiao et al., 2021). The suppression of PPNs by rhizobacteria occurs through different mechanisms, including the ability of the microorganisms to compete for the ecological niche, and the production of nematicidal and antimicrobial compounds (eg., antibiotics, toxins, hydrolytic enzymes) (Migunova and Sasanelli, 2021).

Although *Pseudomonas* spp. have been studied as BCAs of different PPN species (Vagelas et al., 2007; Timper et al., 2009; Trifonova et al., 2014), information on *Nacobbus* spp. is limited. Under greenhouse conditions, Lax et al. (2013b) evaluated the effect of the application of *P. protegens* (ex *P. fluorescens*) CHA0 on *N. celatus*. The strain significantly reduced the number of galls and nematode reproduction (about 60%) on tomato plants, indicating that the bacteria affected the root penetration of infective stages. The nematicidal activity of CHA0 and two derived strains (ARQ1, phenotypically equivalent to CHA0, and CHA89, a mutant that does not produce antibiotics or extracellular proteases) was observed by *in vitro* assays (Marro et al., 2013). At 48 h, all three cell suspensions caused significant J2 mortality, reaching values between 63-69%; CHA89 supernatant produced 40% mortality while CHA0 and ARQ1 showed a higher mortality (89 and 92%, respectively) suggesting that the nematicidal action of strain CHA0 would depend, at least in part, on its exotoxins. Some *Pseudomonas* strains, among them *P. protegens* CHA0, are able to produce antimicrobial metabolites such as 2,4-diacetylphloroglucinol (DAPG) and hydrogen cyanide (HCN), which are important for their biocontrol activity (Haas and Défago, 2005).

*Serratia* spp. are present in a wide range of habitats (water, soil, plants and animals) (Grimont and Grimont, 2006) and have diverse ecological functions, including pathogenic and symbiotic associations (Petersen and Tisa, 2013). *Serratia* spp. secrete secondary metabolites and other biomolecules that are fundamental for success in polymicrobial environments (Petersen and Tisa, 2013). Prodigiosin is a secondary metabolite produced by certain species of *Serratia* (including *S. marcescens*) and its role is still unclear, nevertheless it shows numerous effects on other organisms, such as antimicrobial, insecticidal, antifungal and nematicidal activities (Gulani et al., 2012; Suryawanshi et al., 2014; Lin et al., 2020). This red pigment proved to be an alternative option for biological control of *N. celatus* (Gomez Valdez et al., 2022). *In vitro* experiments showed the nematicidal action of prodigiosin, at low doses, on J2 of two nematode populations, achieving 100% mortality at 48 h. Lethal doses (LD) were obtained (LD_50 =_ 12.4-13.2 μg/mL, LD_90 =_ 24.9 μg/mL) which were then considered for *in vivo* studies. When the metabolite was applied to soil at LD_50_ and LD_90_ doses, it was observed that it reduced the J2 penetration in tomato roots by 59 and 83%, respectively.

### Entomopathogenic nematodes and their symbiotic bacteria

EPNs are among the best BCAs for numerous insect agricultural pests (Campos-Herrera, 2015). The infective juveniles (IJ) of genera *Steinernema* and *Heterorhabditis* are associated with the intestinal symbiotic bacteria *Xenorhabdus* spp. and *Photorhabdus* spp., respectively, that play an important role in host infection and death. The use of EPNs have many attributes, such as being safe for the environment and non-target organisms (Ehlers, 2005).

Several studies have shown an antagonist effect of EPNs on different PPN species, mainly *Meloidogyne* spp. (Kepenekci et al., 2016; Sayedain et al., 2021; El Aimani et al., 2022). The first study with NSC was performed by Caccia et al. (2013). The inoculation with IJs of two Argentinean isolates of EPNs showed an antagonistic action on *N. celatus* multiplication rate in tomato roots, which decreased by 57 and 53% in plants treated with *S. rarum* and *H. bacteriophora*, respectively. It is important to point out that the EPN dose applied in that assay was the one commonly used for insect control in the field (Georgis and Hague, 1991). One of the advantages of using EPNs is that these organisms are commercially available for the management of several insect pests in different countries (Said et al., 2015) and could be used simultaneously for PPN and insect pest management.

In the last few years, emphasis has also been placed on the isolation of symbiotic bacteria because of their promising use in agriculture (Machado et al., 2020). Their metabolites have great biotechnological potential due to insecticidal (da Silva et al., 2020) and nematicidal actions (Kenney and Eleftherianos, 2016). Lax et al. (2013c) showed that *X. szentirmaii* (the symbiont of *S. rarum*) had nematicidal action on J2 and eggs of *N. celatus* under *in vitro* conditions. Furthermore, its application in soil (sterile and unsterilized) with parasitised tomato plants decreased the level of root damage and *N. celatus* reproduction. Posteriorly, Caccia et al. (2018) performed a comparison among different inoculation alternatives of the EPN-bacterial symbiont complex considering other native EPN isolates (*H. bacteriophora*, *Steinernema* sp. and *S. rarum*), their symbiotic bacteria (*P. luminescens*, *Xenorhabdus* sp. and *X. szentirmaii*) and cell-free supernatants. The IJ inoculation did not have a significant effect on the two *N. celatus* populations tested (RC and LUL) on tomato plants while the bacterial suspensions showed different responses. *Xenorhabdus* sp. did not affect the PPN populations whereas *X. szentirmaii* reduced the number of galls (21-28%), and egg masses (25% only in RC). While the *P. luminescens* strain was the most effective against the RC population with a reduction in the number of galls (23%), egg masses (47%) and multiplication rate (63%). Despite the variable results observed with the cell suspensions, a significant antagonist action was produced by the cell-free supernatants on nematode reproduction, especially caused by metabolites of *Xenorhabdus* sp. and *P. luminescens* (62-77%). These results demonstrate the importance of testing different *Nacobbus* populations that may show physiological variability as well as different EPN isolates that may differ in their pathogenicity.

### Mycorrhizal fungi

Arbuscular mycorrhizal fungi (AMF) promote sustainable agriculture due to their role as natural biofertilizers (Smith and Smith, 2011; Baum et al., 2015; Berruti et al., 2016) and bio-protectants against different plant pathogens (Veresoglou and Rillig, 2012; Yang et al., 2014; Hao et al., 2019). Despite mycorrhizae being beneficial in regulating endoparasitic (eg. *Meloidogyne*, *Heterodera*, and *Globodera*) and ectoparasitic PPN species (eg. *Tylenchorhynchus*) (Gough et al., 2020), their mechanisms of action remain largely unknown. AMF are known to improve host tolerance or resistance in many plant-nematode systems and induce systemic resistance against the parasite in roots (Elsen et al., 2008). Their efficacy may be influenced by several factors including environmental conditions (Schwob et al., 1999), cultivar (Masadeh et al., 2004), nutrient status of the field (Waceke et al., 2002), and the timing of application (De La Peña et al., 2006).

Although complex mechanisms underlie the AMF-mediated biocontrol, the AMF inoculation could be easily adopted by horticultural producers. Despite the importance of *Nacobbus* spp. in agriculture, few studies have been carried out so far with AMF, considering mostly nematode Argentinean populations and only tomato and pepper as hosts. The first report indicated that tomato plants inoculated with *Glomus* sp. showed a lower number of galls induced by a Mexican population of *N. aberrans s.l.* (Gardezi et al., 1995). More detailed studies were subsequently conducted on the interaction between *Rhizoglomus intraradices* (ex *Rhizophagus intraradices*) and *N. celatus* in Argentina, showing a significant increase in mycorrhizal colonization (14-55%) in parasitised tomato plants and no consistent results when evaluating plant development parameters (Lax et al., 2011b; Marro et al., 2014). AMF incorporation was beneficial in reducing nematode-induced root damage (lower number of galls), however, the most significant effect was observed on parasite reproduction, which decreased (about 47-69%) in plants treated with AMF at the time of transplanting or three weeks before. These results confirmed beneficial effects when applying AMF at/before transplanting, as a tool to manage nematode populations on tomato.

So far, information about the effect of the inoculation of two or more AMF species on PPNs is limited (Pandey, 2011; Banuelos et al., 2014). Marro (2018) tested the individual and combined application of *R. intraradices* and *Funneliformis mosseae* in parasitised tomato and pepper plants. Damage and multiplication of *N. celatus* decreased significantly in both hosts treated with single or dual AMF species and benefits on biomass were obtained by the AMF combination. More recently, information about the effect of the interaction between *N. aberrans s.l.* and AMF on pepper plant physiology and biochemistry was described (Bernardo et al., 2020). These authors analysed the potential of three AMF (*F. mosseae, R. intraradices* B1 and A2) in the nematode control. Data showed that mycorrhizal association between pepper plants and the tested fungi isolates not only reduced the parasite population but also increased plant mineral nutrition and defense. Besides, *R. intraradices* B1 showed to be the most promising candidate.

One of the antagonistic mechanisms of AMF *vs* PPNs appears related to the alteration of the root metabolism, along with a modified molecular composition of secretions released (Hol and Cook, 2005; Vos et al., 2013). Marro et al. (2018) reported the action of mycorrhizae on *N. celatus* penetration in host roots. Forty five days after the individual and dual application of AMF (*R. intraradices* and *F. mosseae*) on tomato plants, J2 were inoculated and roots were analysed 4, 8 and 12 days after nematode inoculation to evaluate the level of invasion. Plants treated with AMF presented a lower number of juveniles (20-26%) inside the roots compared with non-mycorrhizal plants. However, no synergistic effect against the PPN was observed by applying the two fungi simultaneously. The same action of AMF on J2 penetration was previously reported in *M. incognita* (Vos et al., 2012). Root exudates guide nematodes to the host (Curtis et al., 2009), and the alterations produced by AMF may negatively affect root invasion by the *N. celatus* J2. Since the life cycle of *Nacobbus* spp. has other infective stages (J3, J4 and immature females), it appears important to evaluate the effect of AMF on those stages too.

### Nematophagous fungi

Nematophagous fungi (NF) have a great potential to reduce nematode populations for different reasons: *i)* their superposition with the PPN ecological niche (Yang et al., 2007); *ii)* the capacity of attacking, killing and digesting nematodes (Verdejo-Lucas et al., 2002); *iii)* some of them are facultative saprotrophs, attack other fungi, colonize plant roots or live as endophytes; *iv)* they can be massively produced (Olivares-Bernabeu and López-Llorca, 2002).

*Purpureocillium lilacinum* (ex *Paecilomyces lilacinus*) is a ubiquitous and naturally occurring soil saprotrophic species with antagonistic activity on eggs and females of PPNs (Lamovšek et al., 2013). Its potential in PPN biocontrol was studied on a wide range of species (Brand et al., 2010; Ganaie and Khan, 2010; Kepenekci et al., 2018; Cardona et al., 2020), but few studies have been performed with the NSC. The biocontrol potential of *P. lilacinum* on *N. aberrans s.l.* was first demonstrated, observing parasitised eggs and females in *in vitro* studies (Mareggiani and Gallardo, 1983), and reporting lower number of galls (26.5%) and nematode reproduction (35.5%) in tomato plants under field conditions (Mareggiani et al., 1985). Moreover, 71% of egg masses were infected at the end of the experiment. Another *in vitro* study involving *P. lilacinum* Argentinian isolates (LPSC# 876, Ls and Pv) showed antagonism against eggs of *N. aberrans s.l.*, regardless of their embryonic state (Gortari and Hours, 2019). Hyphae surrounding the eggs were observed after 24 h, and the infection signs (invasion of hyphae, vacuolated, with deformed edges and without remains of the embryo and/or juvenile) became evident at 48 h. Meanwhile, conidiophores and conidia development took place between 48-72 h of culturing. After 5 days, a high proportion of eggs (80-100%) showed variable signs of infection, while the hatching decreased in treated plates by 87-100%, during the evaluation period.

The chitinases and proteases produced by NF could be involved in the decay of nematode eggshells (Tikhonov et al., 2002; Park et al., 2004). The production of secondary metabolites can also be a mechanism of control or suppression of PPNs. Sosa et al. (2018) selected three NF isolates (SR38, SR7 and SR14) with the highest levels of egg (>70%) and J2 (>60%) parasitism in *N. celatus.* They concluded that the most frequent and effective adherence mechanisms were through adhesive hyphae, followed by hyphal networks and adhesive conidia. In some interactions, combined infection mechanisms provided higher parasitism efficiency. Recent studies showed the capacity of these isolates to produce extracellular enzymes and mycotoxins on inductive culture media, and elucidated the mechanisms involved in nematode parasitism (Girardi et al., 2022). The maximum chitinolytic activity (from 0.12 to 0.18 U/h mL) was reached at 13 days of incubation (150 rpm, 0.99 a_W_, 25°C), regardless of the isolate, but protease production significantly differed between them (SR7 = 0.38; SR14, SR38 = 0.15 U/min mL), regardless of the incubation time. Moreover, the production of mycotoxins by isolates ranged between 875.77-2784.91 ng/mL and 227.17-765.39 ng/mL for A and B leucinostatins, respectively. The chitinase and protease production appear related to the nematophagous activity (Park et al., 2004).

Aspects related to multiplication and production conditions of BCAs are important for the development of best application methods. *In-plant* studies with *P. lilacinum* showed that the effectivity on the *N. aberrans s.l.* population differed, depending on the type of substrate evaluated. Tomato inoculated with conidia produced on rice bran and oyster mushroom pomace showed the least number of galls, egg masses and eggs per egg mass, although a conclusive antagonistic activity on the nematode was not evidenced (Gortari and Hours, 2016). Eguiguren-Carrión (1995) also tested, under greenhouse conditions, the *P. lilacinum* potential by applying different doses of colonized rice in tomato and determined the LD_50_ (55.3 mg of colonized rice/500 cm^3^ soil) for an inoculum of 25 J2/cm^3^ soil. On the other hand, the establishment of a fungus with biocontrol potential in the field depends largely on its ability to survive and proliferate in the soil (Magan, 2001). Isolates of *P. lilacinum* SR38, SR7 and SR14 were able to develop saprotrophically in the rhizosphere of tomato (10^4^ cfu/g soil), with SR38 also endophytic in roots (Girardi et al., 2021). In particular, SR14 inoculation incremented plant growth (root and aerial part) with a 47% reduction of the *N. celatus* egg masses (Sosa et al., 2021a).

*Pochonia chlamydosporia* (ex *Verticillium chlamydosporium*) is a widely distributed facultative parasite of nematode egg and root endophyte (Lopez-Llorca et al., 2002; Kerry and Hirsch, 2011; Ceiro-Catasú et al., 2021). Studies with this species on NSC were carried out in Mexico. Several surveys were conducted in sites with different land use (i.e. natural and secondary forests, pasture and cultivated fields) to select those isolates with the highest potential to parasitise eggs of *N. aberrans s.l.* (Flores-Camacho et al., 2008; Franco-Navarro et al., 2008; Franco-Navarro et al., 2012). According to *in vitro* studies, levels of eggs parasitism (59-89%) appeared to depend on the *P. chlamydosporia* isolate and *Nacobbus* population interactions (Flores-Camacho et al., 2008; Franco-Navarro et al., 2012), but not on the substrate used to produce the NF (potato dextrose agar: 77-89% and rice: 72-87%) (Pérez-Rodríguez et al., 2007). Comparative *in vivo* studies on the parasitic capacity of Mexican (MHCH, SM4, SMB3, SMB3A, SC1) and Brazilian (VC10) isolates of *P. chlamydosporia* showed high levels of egg parasitism of populations associated with bean (up to 92%) and tomato (up to 96%) (Flores-Camacho et al., 2007). Meanwhile, the highest egg hatching inhibitions were observed for the interaction between isolates SMB3A (98.5%) and MHCH (91.2%) with tomato-associated populations from Mexico and Puebla, respectively, and nematodes grown in bean (SMB3, SMB3A: 100%). Despite the high percentage of egg parasitism (> 60%), the number of galls and eggs per mass generally increased in presence of the NF, regardless of host-*Nacobbus* populations evaluated. However, assays performed with different *P. chlamydosporia* isolates applied on tomato (Pérez-Rodríguez et al., 2007) and bean (Franco-Navarro et al., 2012) reduced nematode gall indices by 44.1% and 77.3%, J3 and J4 by 96.2% and 77.3%, and mature females by 82.8% and 33.3%, respectively, lowering the nematode damage in both cultures.

*Metarhizium* spp. are entomopathogenic fungi, intensively used in Europe as BCAs (Vivekanandhan et al., 2022). Sosa et al. (2018) reported that *M. robertsii* SR51 and *Plectosphaerella plurivora* SRA14 could parasitise *N. celatus* eggs (85 and 72%, respectively), and J2 (77 and 78%, respectively). The fungus was reported to rely on adhesive conidia and hyphae in the host infection processes. However, the biocontrol activity of *M. robertsii* SR51 did not appear stable, since the infective capacity on J2 did not appear consistent in time.

Nematode trapping fungi are nematode-predatory facultative species living as saprotrophs in soil, that represent a dominant group in the control of PPNs since they produce adhesive net-shaped structures or constricting rings, specially designed for trapping nematodes (Mendoza-de Gives and Torres-Acosta, 2012). Successful assessments were reported for different PPNs, such as *Meloidogyne* spp. (Jansson et al., 1985; Singh et al., 2007; Hastuti et al., 2021). A Mexican isolate of *Arthrobotrys conoides* tested *in vitro* against *N. aberrans s.l.* showed a high trapping capacity (>90% of tested J2), causing total or partial nematode destruction (Mendoza-de Gives et al., 1994). A biocontrol activity was also reported for isolates of the genera *Fusarium* and *Penicillium* from Mexico (Cortez-Hernández et al., 2019). Greenhouse tests on pepper showed that *P. janthinellum* Pe11 reduced root galling (9-49%) and the number of J2 per g of roots (35-56%). Biopesticide formulations based on NF have shown to be effective to control PPNs in various crops worldwide (Sosa et al., 2021b). Three commercial formulates based on *P. lilacinum* (Nemaroot^®^, BioAct Prime^®^, Nematicida PI^®^) and the chemical nematicide fluensulfone (Nimitz 480 EC^®^) were evaluated against *N. aberrans s.l.* on tomato plants (García-Velasco and Chavarro-Carrero, 2020). The bio-nematicides drastically reduced the galling index, the number of egg masses and eggs per g of root of the PPN. Additionally, the control level was higher as the spore concentration of the product increased, which was reflected in their biological effectiveness (76.6-90.1% *vs* 61.7% with fluensulfone).

### Essential oils

These are natural volatile substances, including complex mixtures of terpenoids (monoterpenes and sesquiterpenes) and a variety of aromatic phenols, oxides, ethers, alcohols, esters, aldehydes, and ketones. The EOs are known to affect the permeability of cell membranes, making them susceptible to more toxic components. In addition, depending on the exposure dose, they could interfere with cellular respiration and cause cell lysis (Pérez-Alfonso et al., 2012). Furthermore, they have the advantage of being biodegradable, with a low toxicity.

Nematicidal components have been reported in a variety of plants (Fe Andrés et al., 2012), mainly from the Asteraceae family (Mwamba et al., 2021). Several EOs have been tested for management of *Nacobbus* spp. in different assays. *In vitro* nematicidal effects on *N. celatus* J2 were observed for EOs from oregano (*Origanum vulgare*) and anise (*Pimpinella anisum*) (Sosa et al., 2020). Carvacrol (40%) and thymol (28%) were the main compounds of oregano oil, killing 100% of J2 after 24 h (dose: 600 μL/L). Meanwhile, anise EO killed 100% of J2 after 24 h (dose: 200 μL/L). Chemical analyses revealed high levels of anethole (89.5%), whose nematicidal activity was previously described (Ntalli et al., 2010). *In vitro* assays conducted by De Lillo (2019) showed that the application of english mint (*Mentha piperita*), sweet bay (*Laurus nobilis*), eucalyptus (*Eucalyptus globulus*) and cinnamon (*Cinnamomum verum*) EOs on soil had a nematicidal effect on *N. aberrans s.l.* juveniles (98-100%), after a 24 h exposure. Besides, tomato plants drenching with english mint and eucalyptus EOs showed a lower number of eggs and galls. No signs of phytotoxicity were observed and the best yield was obtained with the application of eucalyptus oil. EO effects on nematode reproduction were also reported in field tests (Rípodas, 2017). Chard (*Beta vulgaris*) plants drenching with mint, sweet bay and eucalyptus solutions (at 6.5% oil concentration) showed a significant reduction of the egg numbers in roots, thus affecting the inoculum remaining in soil in the following spring-summer crop.

Great attention was given to the genus *Tagetes*, which stands out for its allelopathic potential, allowing plants immunity for *Meloidogyne* spp. (Marahatta et al., 2012). Under greenhouse conditions, the application of Mexican marigold (*T. lucida*) oil inhibited the galling induced by *Nacobbus s.l.* on tomato plants, demonstrating that the concentration was a critical factor (LD_50_ = 0.06-0.13 mg/mL) while the application intervals were ineffective. The main compounds of this EO were geranyl acetate, β-ocimene, nerolidol, β-cubebene and caryophyllene (Zarate-Escobedo et al., 2018).

### Plant extracts

Plants synthesize different secondary metabolites, also involved in defense mechanisms. Several experiments were hence conducted against the NSC by testing concentrated, botanical extracts. Nematicidal properties of eucalyptus, castor oil (*Ricinus communis*), chinaberry tree (*Melia azedarach*) and aqueous extracts (AEs) of trichilia (*Trichilia glauca*) were observed on *N. aberrans s.l.* after 48 h exposure. The latter two extracts showed a higher nematicide activity (50% mortality) (Mareggiani et al., 2005). Exposure of *N. aberrans s.l.* J2 to different AE concentrations of lettuce (*Lactuca sativa*), tarwi (*Lupinus mutabilis*) and wild tarwi (*L. chlorilepis*) showed that the last extract had the highest effects, at 40% concentration (mortality after 15 min = 96.7%, after 30 min = 100%) (Velasquez Pari, 2013).

Nematicidal and nematostatic activity of methanolic extracts of endemic plants from the Oaxaca state (Mexico) were also studied at different doses (Velasco-Azorsa et al., 2021) and their effective concentrations (EC, 50% J2 immobility) were estimated. At 48 h, extracts of *Adenophyllum aurantium* (root: EC_50 =_ 62.3-88.3 µg/mL; stem: EC_50 =_ 31.5-110.4 µg/mL), *Alloispermum integrifolium* (EC_50 =_ 47.4-107.1 µg/mL) and *Tournefortia densiflora* (root: EC_50 =_ 59-112.3 µg/mL) showed the highest nematostatic potential on J2 of *N. aberrans s.l.* Extracts of *Alcalypha cuspidata*, *Galium mexicanum*, *Heliocarpus terebinthinaceus* and *T. densiflora* (stem) showed the best nematicidal effects (87-95.4%) at 1000 µg/mL. Despite the high nematostatic activity observed in *A. cuspidata*, *A. subviscida* and *T. densiflora* (stem) extracts, hormetic effects were evidenced, since the germination of tomato seeds cv. Sheva was totally inhibited at 20 μg/mL, while the dose of 0.02 μg/mL promoted the culture growth.

The pesticidal property of the false arnica (*Heterotheca inuloides*) is associated with the high proportion of cadinene-type sesquiterpenes, quercetin and kaempferol type flavonoids (Rodríguez-Chávez et al., 2017). Acetone extract of arnica flowers inhibited eggs hatching of *N. aberrans s.l.* (inhibition at 50%, IC_50 =_ 57.59 and 40.58 mg/L, for eggs with and without gelatinous matrix, respectively) and J2 mobility (LD_50 =_ 32.62 mg/L) (Rodríguez-Chávez et al., 2019). These authors also reported a nematicidal activity of cadinenes.

Studies involving ethyl acetate extract of moringa (*Moringa oleifera*) leaves showed a 90.7% inhibition of *N. aberrans s.l.* egg hatching at a concentration of 5 mg/mL, 12 h after treatment, with 100% J2 mortality achieved at 10 mg/mL, 24 h after treatment (Páez-León et al., 2022). Sosa et al. (2022) reported high mortalities of *N. celatus* J2 exposed to broccoli (*Brassica oleracea* var. *italica*) (LD_100 =_ 250 μL/mL) and cabbage (*B. oleracea* var. *capitata*) (LD_100 =_ 500 μL/mL) AEs, with higher effectiveness at higher doses and longer exposure time.

Despite the promising results obtained through *in vitro* studies, there is a lack of information on the impact of plant extracts on *Nacobbus* populations and host cultures at the greenhouse or field levels. Mareggiani et al. (2005) tested different AEs on pepper infested with *Nacobbus s.l.* Plants treated with chinaberry tree extracts showed a major nematode control with low gall numbers, compared to the other AEs considered (eucalyptus, castor oil and trichilia). Furthermore, no damage against non-target soil organisms (Annelida: Oligochaeta: *Eisenia foetida* and *Dendrobaena octaedro*) was shown.

### Phytohormones

The nematode-host interaction evolved through different defense mechanisms deployed by plants, based on physical barriers (cell wall) and/or an arsenal of molecules and pathways that impede the progression of the parasite invasion. At the molecular level the nematode invasion induces the activation of a host innate immune system response that, according to the plant genotype, activates basal and host-specific responses (Zheng et al., 2021). Plant sophisticated defense mechanisms include the pathways of phytohormones, salicylic and jasmonic acid, whose genes have been characterized in local and systemic responses in many cultivated species associated with *Nacobbus* spp. (Godinez-Vidal et al., 2010; Godinez-Vidal et al., 2013; Villar-Luna et al., 2015; Cabrera-Hidalgo et al., 2021).

Data concerning the effect of plant hormone applications on nematode parasitism were reported by Martinez-Medina et al. (2017). These authors, studying the defense pathways induced by *Trichoderma* spp. for resistance to *M. incognita*, showed that the application of exogenous hormones affects plant defenses by inducing changes on the nematode performance (invasion, damage and reproduction). Recently, 2021; Martinez et al. (2020) reported the impact of the exogenous application of hormones on tomato defenses from *N. aberrans s.l.* Plants grown on nematode infested and hormone-drenching substrate showed, after 60 days, better physiologic parameters than untreated ones, when subjected to salicylic acid (0.5 - 1 × 10^-4^ M), jasmonic acid (10^-4^ - 10^-5^ M) and ethylene (0.35 - 0.70 × 10^-3^ M). Interestingly, plants treated with exogenous application of 10^-5^ M salicylic acid, 24 h before transplanting into infested soil, did not differ from plants grown on an uninfested substrate (Martínez et al., 2020). In addition, the hormone effect as elicitors of resistance to *Nacobbus s.l.* was corroborated by a significantly lower number of galls, gall index, nematode reproduction and increased the total yield of tomato plants, with salicylic acid being the most effective (10^-4^ M) (Martinez et al., 2021).

### Amendments

Use of green and animal manures is a traditional cultural practice improving soil fertility and structure, also used for crop protection. The decomposition of such organic amendments may increase the number of antagonistic microorganisms. Nematicidal metabolites are released changing the physical and biochemical properties of soil, favoring crop development. However, for a successful management of *Nacobbus* spp. with organic residues, it is necessary to conduct previous evaluations in order to define the optimal dose of each amendment and the ideal moment for its incorporation, to reduce as much as possible the nematode damage on crops, and prevent possible phytotoxicity effects (Franco-Navarro et al., 2002).

A reduced number of plants known to contain nematicidal compounds have been used as soil amendments for *Nacobbus* control, in small-scale experiments. Ground seed beans (*Concanavalia ensiformis* and *Mucuna deeringiana*) added to tomato grown in sterilized potting soil (2 or 4 g/pot), one day before *N. aberrans s.l.* inoculation was generally ineffective in reducing the number of galls. However, co-culture of tomato and these legumes showed a significant reduction in root symptoms (Marbán-Mendoza et al., 1989). Gall reduction was attributed to the passage of concanavalin A lectin from *C. ensiformis* into the soil which would interfere with the nematode’s host recognition mechanisms.

Brassicas applied in the soil in various forms (Fourie et al., 2016) have biofumigant properties due to the presence of glucosinolates within their tissues. Effectiveness of biofumigation in soil covered with black polyethylene and incorporated with brown mustard (*B. juncea*) at 2 kg/m^2^, was evaluated on soil nematofauna, including *N. aberrans s.l.* (D´Amico et al., 2019). No significant difference was observed between treatments on PPN densities, but the practice increased the number of free-living nematodes. Under greenhouse conditions, Mitidieri et al. (2009) also did not observe an immediate effect on the nematode population when evaluating different treatments, such as the biofumigation with chicken manure/broccoli and broccoli/turnip (*Brassica napus*). However, 24 months later, significant decreases were observed in the soil population and the number of galls on tomato roots as well as an increase in yields. In the greenhouse, Villa-Briones et al. (2008) also reported a reduction in *N. aberrans s.l.* galling on tomato when vermicompost (27%) and manure (40%) were incorporated, with a biomass increase. In the field, the application of 300 and 500 g/plant of vermicompost decreased root damage by 29%, increasing foliage dry weight (85% and 92%, respectively).

Garita (2019) and Duarte Rolla (2018) evaluated the use of crushed cabbage amendment as biofumigant (140 and 280 g/kg substrate) on tomato. Increased *N. aberrans s.l.* suppression was shown at high amendment concentrations. Although a favorable effect on tomato growth was observed by those authors, proline and malondialdehyde were detected in root tissues of biofumigated plants, possible indicators of phytotoxicity (Garita, 2019). In greenhouse studies, Franco-Navarro et al. (2002) also reported that cabbage and oil castor residues (1 and 2%) applied 10 days before tomato transplanting, had phytotoxic effects and reduced the number of galls of *Nacobbus s.l.* (cabbage: 89-88%, castor oil: 63-70%). Amendments incorporated at transplanting favored the plant growth, regardless of the nematode presence/absence and reduced the root damages (cabbage: 36-54%; castor oil: 21-46%). These results were supported by field trials in which the number of galls was reduced by 66, 50 and 24% when incorporating cabbage residues one week before transplanting (3.25 kg/m^2^), while 72, 53 and 29% reductions were estimated with the incorporation at transplant (5.20 kg/m^2^), and 20, 40 and 60 days after transplant, respectively. In addition, this amendment favored tomato growth and production. By incorporating doses of 5.20 and 3.25 kg/m^2^ of cabbage residues close to the transplant date, dry weight increments were registered in root tissue (24 and 21%), and foliage (41 and 32%), respectively. The total and commercial tomato production increased by 62 and 61% when incorporating cabbage (5.20 kg/m^2^) at transplant, and by 51 and 53% when adding the residues (3.25 kg/m^2^) one week before transplant, respectively.

### Resistance

The exploitation of resistant plant genotypes represents a highly sustainable management practice for PPNs. As tomato, pepper and potato are the three main solanaceous hosts of *Nacobbus* spp., the search for resistance has been mainly focused on these vegetables, mostly directed towards native cultivars/varieties, wild species or known plant material provided with resistant genes against other PPNs. Unfortunately, no resistant commercial tomato or pepper cultivars are available up to the present. In tomato, several evaluations have been conducted in order to find some degree of resistance or tolerance to this nematode. Cap et al. (1993) analysed different *Solanum* spp. accessions, including wild tomato species and only some accessions of *S. peruvianum* and *S. chmielewskii* resulted moderately to highly resistant to an Argentinean population of *N. aberrans s.l.* However, in subsequent tests, interspecific hybrids of *S. lycopersicum* × *L. chmielewskii* accessions, and all their parent lines, showed susceptibility to one population from Argentina, and one from Mexico (Veremis et al., 1997). Resistance sources could not be found in any of the exotic tomato accessions tested. Similar results were obtained by Cazares-Alvarez et al. (2019) when evaluating five wild genotypes and the tomato cv. Río Grande.

In spite of the importance of pepper (*Capsicum* spp.) crop worldwide and of its high susceptibility to the NSC, few screenings have been carried out thus far with available plant material. Brunner de Magar (1967) evaluated the behavior of 90 species and varieties of pepper looking for sources of resistance that could subsequently be used as parents for breeding resistant varieties. The highest resistance was found in three accessions of *C. baccatum* var. *pendulum*, distinguished by a lack of galls or by induction of a few, reduced galls. Some level of resistance was also found in *C. annuum* cv. Calchaquí INTA, Keystone Resistant Giant and Calahorra (Sisler and Casaurang, 1983).

Natural resistance (*R*) genes may effectively limit PPN damage to crops in the field. Resistance to *Meloidogyne* spp. is associated with several dominant genes in *C. annuum* (*N*, *Mech*, *Me*) (Davies and Elling, 2015) and tomato (*Mi*) (Iberkleid et al., 2014). Lax et al. (2006b; Lax et al. 2016) evaluated the response of commercial cultivars and experimental lines of pepper, some carrying resistance genes (*N, Me1, Me3* and *Me7*). Unfortunately, all plant material was susceptible to *N. celatus* populations. In tomato, the *Mi* gene did not confer resistance to *N. aberrans s.l.* either (Sisler and Casaurang, 1983; Cap et al., 1993; Veremis et al., 1997).

**Figure 4:**
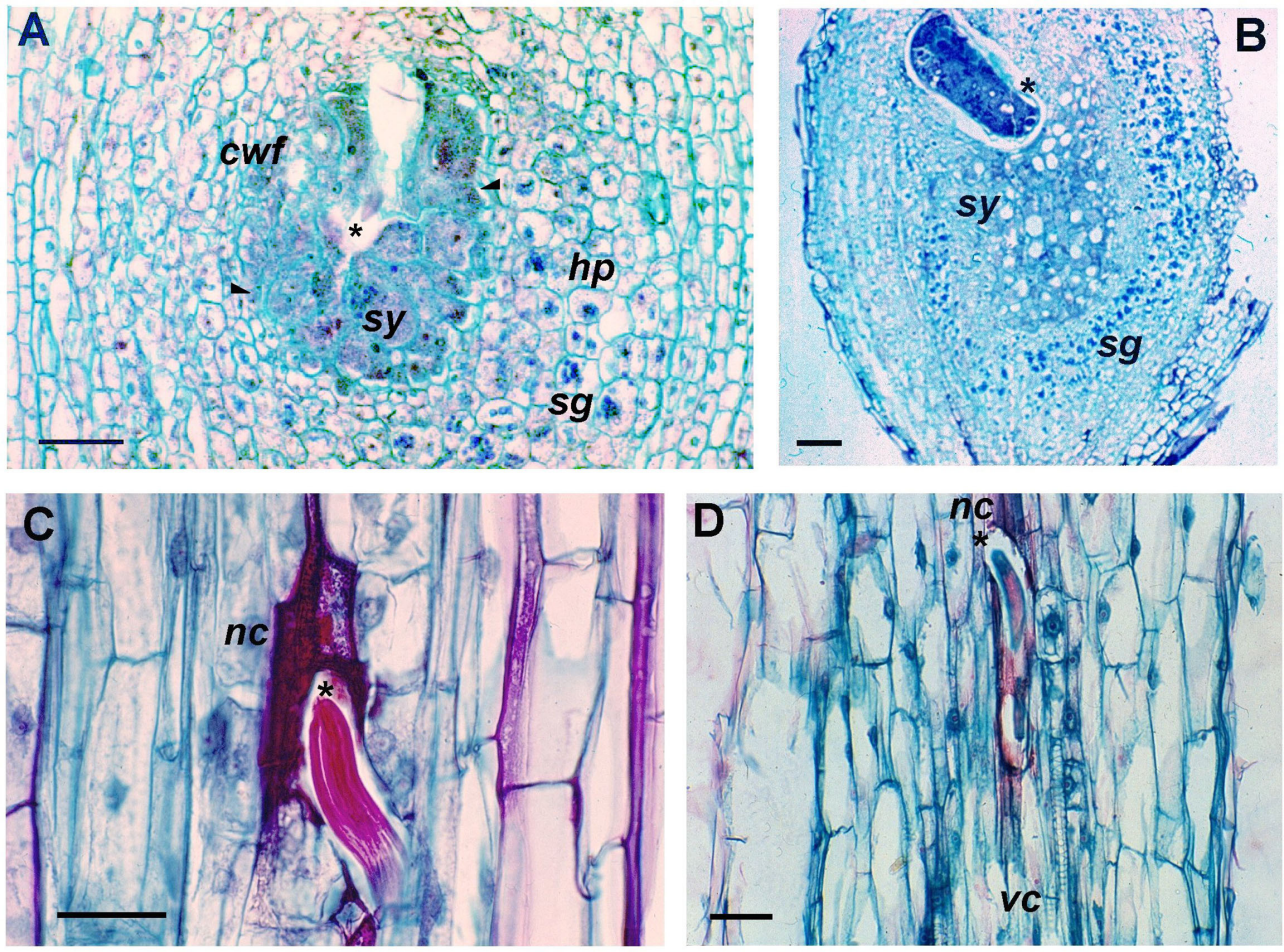
Cytological changes induced by *Nacobbus aberrans s.l.* in potato roots. Longitudinal sections of the susceptible *Solanum tuberosum* cv. Revolución at 10 days after inoculation (dpi) **(A)** and 30 dpi **(B)**, showing the syncytium (*sy*) and the cephalic region of the embedded nematode (asterisk). Syncytium with dense cytoplasm, hypertrophied cells (arrowheads) and nucleoli. Cells with two or more nuclei and cell wall fragments (*cwf*) derived from the cell dissolution. Hyperplasia (*hp*) of the tissue surrounding the syncytium with accumulation of numerous starch grains (*sg*). Longitudinal sections of the resistant hybrid B-25 of *S. tuberosum* Andigenum group, showing the nematode-induced cell necrotic response. At 5 dpi **(C)**, nematode cephalic region surrounded by necrosed cells (*nc*). At 10 dpi **(D)**, starved nematode, adjacent to the vascular cylinder (*vc*), surrounded and trapped by dead cells. Scale bars: A = 200 μm; B–D = 100 μm.

Given their origin in the Andes, potato wild species represent an important source of resistance genes, not only against PPNs but also other plant pathogens. For this reason, many evaluations have focused on local potato genotypes for NSC resistance, mainly in Bolivia and Argentina. The screenings identified genotypes of *Solanum* spp. with different degrees of resistance or susceptibility (see e.g., Alarcón, 1977; Cornejo Quiroz, 1977; Caero, 1985; Costilla, 1985a; Costilla, 1985b; Ortuño et al., 1998; Franco and Main, 2006; Suárez et al., 2009; Tordable et al., 2018). Suárez et al. (2009) tested several cultivated Andean potato landraces that resulted highly susceptible to a potato biotype of *N. aberrans s.l.* from the north-western Argentina, as shown by the production of galls, with only variety Azul showing a lower nematode reproduction. However, the latter variety has been shown to be susceptible to other *Nacobbus* populations from the Andean region (Lax et al., 2008). Wild Andean potato species, including accessions of *S. acaule*, *S. infundibuliforme* and *S. boliviense* (ex *S*. *megistacrolobum*) showed a differential response to *N. aberrans s.l.*, ranging from susceptibility to true resistance, as shown by the 5-10 fold changes found in the number of galls or in the nematode reproduction (Suárez et al., 2009). Among the mechanisms deployed by potato genotypes resistant to the NSC, the early cell necrotic reaction elicited interest as it acts as an initial barrier, activated upon root penetration (Finetti Sialer, 1990; Suárez et al., 2009). In this response, the J2 capability to feed on the invaded tissues is limited by the death of local cells surrounding the nematode. Such a local necrotic reaction does not allow the J2 to complete their life-cycle, nor subsequently induce the gall formation (**Figure 4**). In some situations, it is possible to observe the presence of juveniles surrounded by parenchymatic cells with thick and lignified walls, hindering or restricting nematode movement to the vascular cylinder, where it would establish and subsequently develop a syncytium (Tordable et al., 2018).

To test plant material with resistance/tolerance against other PPNs or pathogens, a good option is also to search for management alternatives. The multiple resistance has fundamental importance, especially for situations where *Nacobbus* spp. co-exist in the same field with other pests such as *Globodera* spp., *Meloidogyne* spp., fungi and viruses. For example, the Huacle chili pepper line 35-3 is resistant to *M. incognita* and *Phytophthora capsici*. It appeared as a potential resistance source into a breeding program for genetic improvement, or as a rootstock for cultivated material susceptible to *N. aberrans s.l*. (Chavarro-Carrero et al., 2017). In Mexico, Leyva-Mir et al. (2013) reported four lines tolerant to *Nacobbus* spp. and some of them also showed tolerance to the other pathogens (*Fusarium oxysporum, Alternaria solani* and *P. infestans*). Screenings conducted using wild potato species resistant to other local Andean pathogens (fungi, insects and viruses) indicated that the highest number of entries resistant to Bolivian *Nacobbus* populations was shown by *S. acaule*, *S. microdontum* and *S. okadae* (63, 83 and 86%, respectively (Franco and Main, 2006). The latter two *Solanum* species were also resistant to *Globodera* spp.

### Integrated strategies

Some of the tactics mentioned above appeared effective at greenhouse or field scales to control NSC. However, a more viable alternative considers their use in a mid to long term integrated management (IM). The IM aim is to achieve a synergistic effect keeping the *Nacobbus* population densities below harmful levels to reduce host damage and yield losses. However, from a field application perspective, it is also important to evaluate not only the varying concentrations of the different alternatives available (such as BCAs, amendments, natural products) but also their timing of application, to define the best, possible sequential strategy within an IM plan.

Crop rotation also plays an important role when combining different management strategies against NSC, because it may contribute to a further decline in the nematode soil population. This effect was reported in a broad bean (*Vicia faba*)-potato system by Iriarte et al. (1999). These authors evaluated the effect of incorporating foliage from a previous broad bean crop (5, 10 and 15 t/ha, row and broadcast application), with cow manure (0, 5 and 10 t/ha). Broad bean crop rotation reduced the *N. aberrans s.l.* initial population by 30%. Moreover, the combination of both amendments at 5 t/ha reduced the nematode multiplication rate, with a higher economic benefit for the farmers. Green manure, however, showed a phytotoxic effect at rates higher than 10 t/ha. Balderrama et al. (1993) tested, under greenhouse conditions, the incorporation in soil of a mixture of different animal manures (cattle, sheep and chicken, at 7 t/ha) previously inoculated with *Beauveria brongniartii* conidia produced on rice or barley. Amendments only reduced the eggs hatching when the fungus was inoculated with sheep manure amendments.

Integration of four control strategies (carbofuran, vermicompost, cabbage fragments, *P. chlamydosporia*) was also evaluated (Pérez-Rodríguez et al., 2011). In greenhouse, pepper plants treated with vermicompost, alone or in combination, showed a lower number of galls (vermicompost/nematicide/cabbage/NF= 46%; vermicompost= 49%; vermicompost/nematicide/NF= 65%), juveniles (93%) and females per g of root, with an increase in dry top plant weight. Interestingly, field trials showed that nematode root damage was moderate to low in those cultures where only vermicompost or cabbage fragments were added. At the end of the trial, lower gall index scores were also recorded (vermicompost/cabbage=70%; vermicompost/NF=73%). In addition, both treatments also decreased by 56% the juveniles in roots. The 25% reductions of females were attributed to the cabbage fragments alone or in combination with vermicompost. Egg masses were colonized by the NF, with 23, 27 and 23% prevalence at 60, 80 and 100 days after transplant, respectively. A similar IM study was carried out by Pérez-Espíndola. (2019), in which biofumigation strategies were combined with *P. chlamydosporia* to improve tomato production in soil naturally infested by *N. aberrans s.l.* and *M. incognita*. NF effectively colonized tomato roots and egg masses. However, the highest effects were found in the treatment with chicken manure/shredded cabbage/NF, with 68% of egg masses infected and 50% and 40% reductions in gall index and J2, respectively.

As mentioned above, the biofumigation with brassicas has been shown to be effective (at appropriate doses) for NSC control. Garita (2019) evaluated biofumigation practices with cabbage on tomato (application at 30, 15, 0 days before transplanting) in combination with AMF (*F. mosseae*). Among the AMF treatments, the population of *N. aberrans s.l.* was lower when the soil was biofumigated 15 days before transplanting. This response appears related to the toxicity that the compounds released during the biofumigation process exert on the AMF and the root mycorrhization. This is consistent with reports by Grosso (2020) who also did not report a synergistic effect against the nematode population when incorporating cabbage at transplant (140 g/kg soil) in tomato plants pre-mycorrhized with *F. mosseae*. Garita (2019) also performed a co-inoculation test between NF (*P. lilacinum* and *Pleurotus ostreatus*) and *F. mosseae* and reported an increase in mycorrhizal colonization in tomato roots, with compatibility between the fungi. No differences in nematode population were observed among treatments (AMF, NF, AMF+NF). However, a nematode control was not included to confirm the efficiency of the different microorganisms. In another study conducted by Molar and Sacks (2021), a suppressive effect on *Nacobbus s.l*. was observed on pepper seedlings treated with different microbial agents, alone or co-inoculated (*P. lilacinum*, *Bacillus thuringiensis* and *R. intraradices*). Co-inoculated BCAs mitigated the plant damages (growth and physiological parameters, as stomatal conductance, net photosynthesis, photosystem II quantum yield, relative conductivity of cell membranes, and soluble protein content) and therefore the stress produced by the nematode, improving plant growth and yield.

Grafting and symbiosis with AMF can also represent effective tools for PPN management. Garita et al. (2019) compared the response of tomato plants cv. Santa Clara with plants grafted on two types of rootstocks (cherry, *S. lycopersicon* var. *cerasiform* Carolina, and Maxifort, carrying the resistant *Mi* gene), combined with *R. intraradices*, in soils infested with *N. aberrans s.l*. The Maxifort rootstock had an enhancing effect on the growth of tomato while the Carolina rootstock showed a reducing effect. Mycorrhizal colonization reduced the number of days until flowering, lowering the final nematode population, on the three plants tested.

The efficacy of biocontrol fungi that live in the soil could be affected by conventional and organic agronomic practices through pesticides (Ondráčková et al., 2019) and botanical extracts (Abarca Antamba and Herrera Zambrano, 2022), respectively. The *in vitro* compatibility of the *P. lilacinum* SR14 was tested with broccoli AE and anise EO to control *N. celatus*. The combined treatment (200 μL/L) induced a higher (86%) J2 mortality (Sosa et al., 2020; Sosa et al., 2022). The broccoli AE increased the growth rate (by 8%) and conidiation (by 27%) of SR14 and its ability to J2 infection (by 76%) (Sosa et al., 2022). This latter combined strategy was also evaluated in tomato plants grown on a sterile substrate under greenhouse conditions, showing a lower gall index and egg masses numbers, as well as a lower nematode reproduction rate for treated plants (Sosa et al., 2021c).

### Perspectives and conclusions

Correct nematode identification and knowledge about the diversity of species, early detection and phytosanitary regulations (including quarantine standards, plant health and certification programs for free plant material of *Nacobbus* spp.) play an important role in the prevention and control of this PPN. Adequate implementation of the restriction measures established by different countries for this parasite is needed not only to prevent the pest introduction, but also to prevent the entry of a particular group/pathotype that is not present in a given region and that may affect a particular crop (Inserra et al., 2005).

Due to the limited availability of cultivars with resistance to *Nacobbus* spp. and given the wide range of variation in parasitism (pathotypes/groups) at species or intraspecific level, knowledge on the nematode population biology is hence fundamental when planning management strategies. However, it is important to highlight that the host range of a population classified as a particular physiological group may be even wider, when compared to the differential hosts. This complicates management, in addition to the nematode’s great adaptive capacity and efficient dispersal mechanisms. To be successful, IM requires a detailed knowledge of the different agro-ecosystems as well as the *Nacobbus*-plant genotypes interactions. The application of effective practices is often difficult, due to the great variability of agricultural, ecological, socio-economic, cultural and political aspects. For this reason, the IM has to be adapted to the crop and region where the nematode problem occurs. It must be efficient, economic and ecosystem-friendly and, as far as it is possible, coincide with the practices that local farmers develop in their farming systems (Franco et al., 1992).

Given the importance of this nematode for agriculture, further studies on IM should be carried out, especially in plans that include: i) crop rotation with hosts, non-hosts and trap plants, ii) different BCAs and/or their metabolites, iii) AEs and EOs from native flora, and iv) compatibility of the BCAs and natural products with nematicides. The use of microbial biocontrol agents appears to be more advantageous than organic amendments because they are generally easier to multiply and apply in soil. The success of amendments depends on applications of appropriate volumes to the soil (sometimes large quantities of plant and/or animal manure are required), and should consider the drawback that, in many cases, they can show phytotoxicity.

Use of microbial biocontrol agents appears more advantageous than the application of organic amendments, given that in the latter case large quantities of plant and/or animal manure are required. The success of organic amendments depends on applications of appropriate volumes to the soil surface, and should consider the drawback that, in many cases, they can show phytotoxicity.

On the other hand, the search for resistance to *Nacobbus* spp. represents a highly sustainable management practice. However, this technology is not always available to farmers, depending on: i) the availability and field performance of suitable plant genotypes, ii) the nematode adaptation and the triggering pathogenicity, overcoming host resistance, and iii) the effect of extra-farm factors, including the market demands for susceptible cultivars and their commercial products. The development and rational use of resistant material requires large and permanent investments of human and economic resources. In addition, farmers and professionals need to be properly trained for their correct use so that they do not apply them indiscriminately. The inappropriate use of this strategy can allow the re-emergence or selection of more aggressive *Nacobbus* group/pathotypes, or change the dominance of other PPN species that naturally coexist in the soil, exacerbating crop damages.

## Author contributions

All authors listed have made a substantial, direct, and intellectual contribution to the work and approved it for publication.

## Funding

PL acknowledges support from the Agencia Nacional de Promoción Científica y Tecnológica (Préstamo BID, PICT 2020 N° 1342) and the Consejo Nacional de Investigaciones Científicas y Técnicas (CONICET) (PIP 11220200101685). MP acknowledges support from the Agencia Nacional de Promoción Científica y Tecnológica (Préstamo BID, PICT 2020 N° 1560) and the Secretaría de Ciencia y Técnica, Universidad Nacional de Río Cuarto (SECYT- UNRC), PPI-2019 Res. 161, 2020-2023.

## Acknowledgments

The authors thanks to Dr. Ana Marisa Matesevach, staff of the herbarium CORD (Museo Botánico de Córdoba), for her disposition to provide information of the Solanaceae taxonomy.

## Conflict of interest

The authors declare that the research was conducted in the absence of any commercial or financial relationships that could be construed as a potential conflict of interest.

## Publisher’s note

All claims expressed in this article are solely those of the authors and do not necessarily represent those of their affiliated organizations, or those of the publisher, the editors and the reviewers. Any product that may be evaluated in this article, or claim that may be made by its manufacturer, is not guaranteed or endorsed by the publisher.
