# Supplementary material for: Sustainable strategies for management of the “false root-knot nematode” Nacobbus spp
Source: Front Plant Sci. 2022 Nov 25;13:1046315. doi: 10.3389/fpls.2022.1046315 (PMC9774502; doi:10.3389/fpls.2022.1046315)
Supplement: Supplementary file 1 [file Table_1.docx]

**TABLE 1 |** Response of *Nacobbus* spp. to the interaction with different biological control agents in *in vitro* experiments.

| **Biocontrol agent (BCA)** | ***Nacobbus* spp. and origin** | **Response to BCA-*Nacobbus* interaction** | **Reference** |  |
| --- | --- | --- | --- | --- |
| **Bacteria** |  |  |  |  |
| *Pseudomonas protegens* CHA0, ARQ1, CHA89 | *N. celatus**; Argentina | J2 mortality (CHA0, ARQ1) | Marro et al. (2013) |  |
| *Xenorhabdus szentirmaii* | *N. celatus*; Argentina | J2 mortality, ↓ egg hatching | Lax et al. (2013c) |  |
| **Nematophagous fungi** | |  |  |  |
| *Purpureocillium lilacinum* (LPSC# 876, Ls, Pv) | *N. aberrans s.l.*; Argentina: Buenos Aires | ↓ Egg hatching | Gortari and Hours (2019) |  |
| *P. lilacinum* (SR38, SR7, SR14) | *N. celatus;* Argentina: Córdoba | ↑ Egg and J2 parasitism  ↑ Chitinase, protease, Leu A and B | Sosa et al. (2018), Girardi et al. (2022) |  |
| *Pochonia chlamydosporia* (MHCH, SM4, SMB3, SMB3A, SC1) | *N. aberrans s.s.*; Mexico: Mexico, Morelos, Puebla and Tlaxcala | ↑ Egg parasitism | Flores-Camacho et al. (2008) |  |
| *P. chlamydosporia* (SC1, IZ1) | *N. aberrans s.l.*; Mexico: Zacatecas | ↑ Egg parasitism | Franco-Navarro et al. (2012) |  |
| *P. chlamydosporia* (MPc1-MPc5) | *N. aberrans s.l.*; Mexico: Puebla | ↑ Egg parasitism | Pérez-Rodríguez et al. (2007) |  |
| *Metarhizium robertsii* (SR51), *Plectosphaerella plurivora* (SRA14) | *N. celatus*; Argentina: Córdoba | ↑ Eggs and J2 parasitism | Sosa et al. (2018) |  |
| *Arthrobotrys conoides* (Montecillo) | *N. aberrans s.l.* | Trapping capacity of J2 | Mendoza-de Gives et al. (1994) |  |
| *Fusarium solani* (Fs03), *F. oxysporum* (Fo07, Fo10), *Penicillium janthinellum* (Pe11) | *N. aberrans s.l.*; Mexico | ↑ Eggs and J2 parasitism | Cortez-Hernández et al. (2019) |  |

* All *N. celatus* populations were previously identified as *N. aberrans*. Abbreviations: J2, second-stage juveniles; Leu, Leucinostatin.
