# Supplementary material for: Sustainable strategies for management of the “false root-knot nematode” Nacobbus spp
Source: Front Plant Sci. 2022 Nov 25;13:1046315. doi: 10.3389/fpls.2022.1046315 (PMC9774502; doi:10.3389/fpls.2022.1046315)
Supplement: Supplementary file 2 [file Table_2.docx]

**TABLE 2 |** Response of *Nacobbus* spp. to the interaction with different biorational products (metabolites, essential oils, extracts) in *in vitro* experiments.

| **Biorational chemical agents** | ***Nacobbus* spp. and origin** | **Conditions tested** | **Action on *Nacobbus*** | **Reference** |
| --- | --- | --- | --- | --- |
| **Bacterial metabolite** |  |  |  |  |
| Prodigiosin | *N. celatus**; Argentina: Córdoba, Catamarca | Direct contact (LD_50, 90_) | J2 mortality | Gomez Valdez et al. (2022) |
|  |  |  |  |  |
| **Essential oils** |  |  |  |  |
| *Origanum vulgare, Pimpinella anisum* | *N. celatus*; Argentina: Córdoba | Direct contact (LD_100_) | J2 mortality | Sosa et al. (2020) |
|  |  |  |  |  |
| *Mentha piperita, Laurus nobilis, Eucalyptus globulus, Cinnamomum verum* | *N. aberrans s.l.*; Argentina: Buenos Aires | Soil application | J2 mortality  No effect on egg hatching | De Lillo (2019) |
|  |  |  |  |  |
| *M. piperita, L. nobilis, E. globulus* | *N. aberrans s.l.*; Argentina: Buenos Aires | Direct contact,  soil application | Nematostatic effect on juveniles | Rípodas (2017) |
|  |  |  |  |  |
| **Extracts** |  |  |  |  |
| *Melia azedarach, E. globulus, Trichilia glauca, Ricinus communis* | *N. aberrans s.l.*; Argentina | Aqueous extracts, direct contact | J2 mortality | Mareggiani et al. (2005) |
|  |  |  |  |  |
| *Lactuca sativa, Lupinus mutabilis, L. chlorilepis* | *N. aberrans s.l*.; Peru: Puno | Aqueous extracts, direct contact | J2 mortality | Velasquez Pari (2013) |
|  |  |  |  |  |
| *Acalypha cuspidata, A. subviscida, Adenophyllum aurantium, Alloispermum integrifolium, Galium mexicanum, Heliocarpus terebinthinaceus, Tournefortia densiflora* | *N. aberrans s.l.*; Mexico | Methanolic extract (EC_50_) | J2 mortality  Nematostatic effect on J2 | Velasco-Azorsa et al. (2021) |
|  |  |  |  |  |
| *Heterotheca inuloides* | *N. aberrans s.l.*; Mexico | Natural and semisynthetic cadinenes, acetone extract (IC_50_; LD_50_) | ↓ Egg hatching  J2 mortality | Rodríguez-Chávez et al. (2019) |
|  |  |  |  |  |
| *Moringa oleifera* | *N. aberrans s.l.*; Mexico | Ethyl acetate extract (leaf tissue) | ↓ Egg hatching  J2 mortality | Páez-León et al. (2022) |
|  |  |  |  |  |
| *Brassica oleracea* var. *italica, B. oleracea* var. *capitata* | *N. celatus*; Argentina: Córdoba | Aqueous extracts (LD_100_) | J2 mortality | Sosa et al. (2022) |
|  |  |  |  |  |

* All *N. celatus* populations were previously identified as *N. aberrans*. Abbreviations: J2, second-stage juveniles; EC_50_, J2 immobility at 50%; IC_50_, inhibition of hatching at 50%; LD, lethal dose.
