# Supplementary material for: Sustainable strategies for management of the “false root-knot nematode” Nacobbus spp
Source: Front Plant Sci. 2022 Nov 25;13:1046315. doi: 10.3389/fpls.2022.1046315 (PMC9774502; doi:10.3389/fpls.2022.1046315)
Supplement: Supplementary file 3 [file Table_3.docx]

**TABLE 3 |** Response of *Nacobbus* spp. to different strategies of management using organic amendments.

| **Amendments** | ***Nacobbus* spp. and origin** | **Cultures tested** | **Action on *Nacobbus* spp.** | **Reference** |
| --- | --- | --- | --- | --- |
|  |  |  |  |  |
| *Concanavalia ensiformis, Mucuna deeringiana* (ground seeds or co-culture) | *N. aberrans s.l*.; Mexico: Chapingo | Tomato cv. Rutgers and Ace | No effect on galls (ground seeds)  ↓ Galls (co-culture)  Inconsistent results on root growth | Marban-Mendoza et al. (1989) |
|  |  |  |  |  |
| *Brassica oleracea* var*. capitata, Ricinus communis* | *N. aberrans s.l*.; Mexico: Puebla | Tomato cv. Río Grande; greenhouse | ↓ Galls  Phytotoxic effect (application 10 d before transplanting)  ↑ Plant growth (application at time of transplanting) | Franco-Navarro et al. (2002) |
|  |  | Field | ↓ Galls  ↑ Plant growth and total yield |  |
|  |  |  |  |  |
| *B. oleracea* var*. capitata* | *N. aberrans s.l.*; Argentina: Buenos Aires | Tomato cv. Platense | ↓ Galls and nematode reproduction  ↑ Plant growth (possible phytotoxic effect) | Duarte Rolla (2018), Garita (2019) |
|  |  |  |  |  |
| *B. oleracea* var. *italica, Brassica napus,* chicken manure | *N. aberrans s.l.*; Argentina: Buenos Aires | Tomato cv. Superman | ↓ Galls and soil population (24 months after biofumigation) | Mitidieri et al. (2009) |
|  |  |  |  |  |
| *Brassica juncea* | *N. aberrans s.l.*; Argentina: Buenos Aires | Incorporation of the amendment into the soil, no culture | No effect on nematode population | D´Amico et al. (2019) |
|  |  |  |  |  |
| *Vicia faba,* cow manure | *N. aberrans s.l.*; Bolivia: Cochabamba | Potato cv. Waych'a | ↓ Galls and nematode reproduction  ↑ Plant yield | Iriarte et al. (1999) |
|  |  |  |  |  |
| Vermicompost; animal manure | *N. aberrans s.l.*; Mexico: Montecillo | Tomato cv. Rio Grande; greenhouse and field | ↓ Galls index  ↑ Plant growth  ↓ Root necrosis | Villa-Briones et al. (2008) |
|  |  |  |  |  |
