# Supplementary material for: Sustainable strategies for management of the “false root-knot nematode” Nacobbus spp
Source: Front Plant Sci. 2022 Nov 25;13:1046315. doi: 10.3389/fpls.2022.1046315 (PMC9774502; doi:10.3389/fpls.2022.1046315)
Supplement: Supplementary file 4 [file Table_4.pdf]

Supplementary references

Cortez Hernández, M. A., Rojas-Martínez, R. I., Pérez-Moreno, J., Ayala-Escoba, V., Silva-Valenzuela, M., and Zavaleta-Mejía, E. (2019). Control biológico de *Nacobbus aberrans* mediante hongos antagonistas. *Nematropica* 49, 140–151.

D'Amico, M., Perniola, O. M., Chorzempa, S. E., Cap, G. B., Castaldo, V., Olaechea, B., et al. (2019). Biofumigación con mostaza parda (*Brassica juncea* L. czern.). Efecto sobre la nematofauna del suelo. 1° Congreso Argentino Agroecología UNCUYO, 466–469.

De Lillo, T. (2019). Aplicación de aceites esenciales en tomate (*Solanum lycopersicum* L.) como alternativa al control de *Nacobbus aberrans* (dissertation/ Degree thesis) (La Plata: Universidad Nacional de la Plata).

Duarte Rolla, M. M. (2018). Efecto de la biofumigación con brassicáceas sobre *Nacobbus aberrans* en plantas de tomate platense (*Solanum lycopersicum* L. var. platense). http://sedici.unlp.edu.ar/handle/10915/70808

Flores-Camacho, R., Atkins, S., Manzanilla-López, R. H., Cid del Prado-Vera, I., and Martínez-Garza, A. (2008). Caracterización de aislamientos mexicanos de *Pochonia chlamydosporia* var. chlamydosporia (Goddard) Gams y Zare para el control biológico de *Nacobbus aberrans* (Thorne) Thorne y Allen. Rev. *Mex. Fitopatol*. 26, 93–104.

Franco-Navarro, F., Cid del Prado-Vera, I., Zavaleta-Mejía, E., and Sánchez- García, P. (2002). Application of organic amendments for the management of *Nacobbus aberrans* on tomato. *Nematropica* 32, 113–124.

Franco-Navarro, F., Cid del Prado-Vera, I., and Romero Tejeda, M. L. (2012). Aislamiento y potencial parasítico de un aislamiento nativo de *Pochonia chlamydosporia* en contra de *Nacobbus aberrans* en frijol. *Rev. Mex. Fitopatol*. 30, 101–114.

Garita, S. A. (2019). Herramientas biológicas (Un aporte para elaboración de un plan de manejo de *Nacobbus aberrans* (dissertation/Doctoral Thesis. La Plata: Universidad Nacional de la Plata).

Girardi, N. S., Sosa, A. L., Etcheverry, M. G., and Passone, M. A. (2022). In vitro characterization bioassays of the nematophagous fungus *Purpureocillium lilacinum*: evaluation on growth, extracellular enzymes, mycotoxins and survival in the surrounding agroecosystem of tomato. *Fungal Biol*. 126, 300–307. doi: 10.1016/j.funbio.2022.02.001

Gomez Valdez, L., Rondan Dueñas, J. C., Andrade, A. J., Del Valle, E., Doucet, M. E., and Lax, P. (2022). In vitro and in vivo nematicidal activity of prodigiosin against the plant-parasitic nematode. *Nacobbus celatus*. *Biocontrol Sci. Technol*. 32, 741–751. doi: 10.1080/09583157.2022.2045474

Gortari, M. C., and Hours, R. A. (2019). In vitro antagonistic activity of argentinean isolates of *Purpureocillium lilacinum* on *Nacobbus aberrans* eggs. *Curr. Res. Environ. Appl. Mycol*. 9, 164–174. doi: 10.5943/cream/9/1/14

Iriarte, L., Franco, J., and Ortuño, N. (1999). Efecto de abonos orgánicos sobre las poblaciones de nematodos y la producción de la papa. *Rev. Latinoam. Papa* 11, 149–163. doi: 10.37066/ralap.v11i1.101

Lax, P., Rondan Dueñas, J., Caccia, M., Marro, N., Becerra, A. and Doucet, M. E. (2013c). Effect of steirnernema rarum and its symbiotic bacterium Xenorhabdus szentirmaii on *Nacobbus aberrans. Nematropica* 43, 305–306.

Marbán-Mendoza, N., Dicklow, M. B., and Zuckerman, B. M. (1989). Evaluation of control of *Meloidogyne incognita* and *Nacobbus aberrans* on tomato by two leguminous plants. *Rev. Nematol*. 12, 409–412.

Mareggiani, G., Zamuner, N., Franzetti, D., Michetti, M., and Collavino, M. (2005). Impact of natural extracts on target and non target soil organisms. *Bol. San. Veg. Plagas* 31, 443–448.

Marro, N., Becerra, A., Valverde, C., Agaras, B., Doucet, M., and Lax, P. (2013). Evaluación in vitro de tres cepas de *Pseudomonas protegens* sobre juveniles de segundo estadio de *Nacobbus aberrans. Nematropica* 43, 309.

Mendoza-de Gives, P., Zavaleta-Mejia, E., Herrera-Rodriguez, D., and Quiróz-Romero, H. (1994). In vitro trapping capability of *Arthrobotrys* spp. on infective larvae of *Haemonchus contortus* and *Nacobbus aberrans*. *J. Helminthol*. 68, 223– 229. doi: 10.1017/S0022149X00014383

Mitidieri, M., Brambilla, V., Saliva, V., Piris, E., Piris, M., Celié, R., et al. (2009). Efecto de distintas secuencias de tratamientos de biofumigación sobre parámetros fisicoquímicos y biológicos del suelo, el rendimiento y la salinidad de cultivos de tomate y lechuga bajo cubierta. *Horticultura Argent*. 28, 5–17.

Páez-León, S. Y., Carrillo-Morales, M., Gómez-Rodríguez, O., López-Guillén, G., Castañeda-Ramírez, G. S., Hernández-Núñez, E., et al. (2022). Nematicidal activity of leaf extract of *Moringa oleifera* lam. against *Haemonchus contortus* and *Nacobbus aberrans*. *J. Helminthol*. 96, e13. doi: 10.1017/S0022149X22000025

Pérez-Rodríguez, I., Doroteo-Mendoza, A., Franco-Navarro, F., Santiago-Santiago, V., and Montero-Pineda, A. (2007). Isolates of *Pochonia chlamydosporia* var. chlamydosporia from Mexico as potential biological control agents of *Nacobbus aberrans. Nematropica* 37, 127–134.

Rípodas, J. I. (2017). Tratamientos no convencionales para el control de *Nacobbus aberrans* en acelga (dissertation/Degree thesis) (La Plata: Universidad Nacional de la Plata).

Rodríguez-Chávez, J. L., Franco-Navarro, F., and Delgado, G. (2019). In vitro nematicidal activity of natural and semisynthetic cadinenes from *Heterotheca inuloides* against the plant-parasitic nematode *Nacobbus aberrans* (Tylenchida: Pratylenchidae). *Pest Managem*. Sci. 75, 1734–1742. doi: 10.1002/PS.5294

Sosa, A. L., Rosso, L. C., Salusso, F. A., Etcheverry, M. G., and Passone, M. A. (2018). Screening and identification of horticultural soil fungi for their evaluation against the plant parasitic nematode *Nacobbus aberrans. World J. Microbiol. Biotechnol*. 34, 63–75. doi: 10.1007/s11274-018-2441-8

Sosa, A. L., Girardi, N. S., Rosso, L. C., Etcheverry, M. G., and Passone, M. A. (2022). In vitro compatibility of Brassicaceae extracts with nematophagous fungi and their effects against *Nacobbus celatus. World J. Microbiol. Biotechnol.* 38, 138. doi: 10.1007/s11274-022-03318-0

Sosa, A. L., Girardi, N. S., Rosso, L. C., Salusso, F., Etcheverry, M. G., and Passone, A. (2020). In vitro compatibility of *Pimpinella anisum* and *Origanum vulgare* essential oils with nematophagous fungi and their effects against *Nacobbus aberrans*. *J. Pest Sci.* 93, 1381–1395. doi: 10.1007/s10340-020-01252-4

Velasco-Azorsa, R., Cruz-Santiago, H., Cid del Prado-Vera, I., Ramirez-Mares, M. V., Gutiérrez-Ortiz, M. R., and Santos-Sánchez, N. F. (2021). Chemical characterization of plant extracts and evaluation of their nematicidal and phytotoxic potential. *Molecules* 26, 1–13. doi: 10.3390/molecules26082216

Velasquez Pari, M. (2013). Extractos de plantas con potencial nematicida en el control del falso nematodo del nudo de la raíz (*Nacobbus* spp.) Universidad Nacional del Altiplano, Puno, Perù.

Villa-Briones, A., Zavaleta-Mejía, E., Vargas-Hernández, M., Gómez-Rodríguez O., and Ramírez-Alarcón, S. (2008). Incorporación de vermicomposta en el manejo de *Nacobbus aberrans* en jitomate (*Lycopersicon esculentum* mill.). Rev. Chapingo Ser. Hortic. 14, 249–255.
